# 4-(4-Bromo­phen­yl)-2-methyl­amino-3-nitro-5,6,7,8-tetra­hydro-4*H*-chromen-5-one

**DOI:** 10.1107/S1600536813012774

**Published:** 2013-05-22

**Authors:** P. Narayanan, Jayabal Kamalraja, Paramasivam T. Perumal, K. Sethusankar

**Affiliations:** a Department of Physics, RKM Vivekananda College (Autonomous), Chennai 600 004, India; b Organic Chemistry Division, Central Leather Research Institute, Adyar, Chennai 600 020, India

## Abstract

In the title compound, C_16_H_15_BrN_2_O_4_, the six-membered carbocyclic ring of the chromene moiety adopts an envelope conformation with the disordered methyl­ene C atom as the flap. The pyran ring is almost orthogonal to the chloro­phenyl ring, making a dihedral angle of 87.11 (12)°. The amine-group N atom deviates significantly from the pyran ring [0.238 (3) Å]. The mol­ecular structure is stabilized by an intra­molecular N—H⋯O hydrogen bond, which generates an *S*(6) ring motif. In the crystal, mol­ecules are linked *via* C—H⋯O hydrogen bonds, which generate *C*(8) chains running parallel to the *b* axis. The chains are linked by C—H⋯π inter­actions. The methyl­ene-group C atom of the chromene system that is disordered, along with its attached H atoms and the H atoms on the two adjacent C atoms, has an occupancy ratio of 0.791 (7):0.209 (7).

## Related literature

For the uses and biological importance of chromene, see: Ercole *et al.* (2009 ▶); Geen *et al.* (1996 ▶) Khan *et al.* (2010 ▶); Raj *et al.* (2010 ▶). For a related structure, see: Sun *et al.* (2012 ▶). For graph-set notation, see: Bernstein *et al.* (1995 ▶).
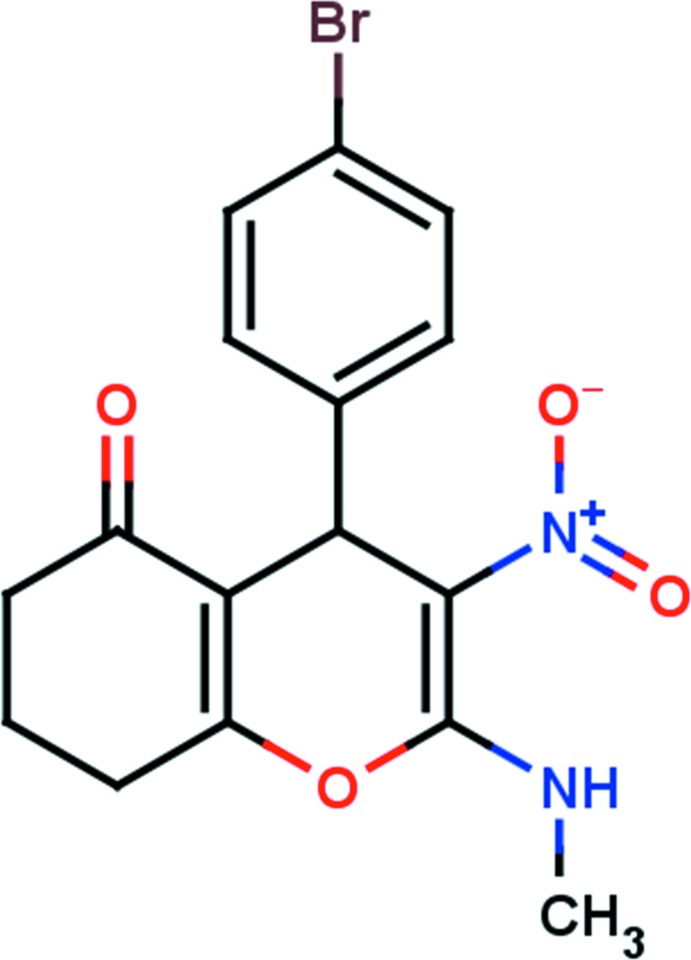


## Experimental

### 

#### Crystal data

C_16_H_15_BrN_2_O_4_
*M* *_r_* = 379.18
Monoclinic, 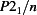

*a* = 8.1114 (9) Å
*b* = 10.8530 (13) Å
*c* = 18.222 (2) Å
β = 94.399 (6)°
*V* = 1599.4 (3) Å^3^
*Z* = 4
Mo *K*α radiation
μ = 2.59 mm^−1^
*T* = 296 K
0.30 × 0.25 × 0.25 mm

#### Data collection

Bruker SMART APEXII CCD diffractometer
Absorption correction: multi-scan (*SADABS*; Bruker, 2008 ▶) *T*_min_ = 0.464, *T*_max_ = 0.523
12198 measured reflections
3130 independent reflections
2053 reflections with *I* > 2σ(*I*)
*R*_int_ = 0.047

#### Refinement

*R*[*F* ^2^ > 2σ(*F* ^2^)] = 0.039
*wR*(*F* ^2^) = 0.101
*S* = 1.04
3130 reflections
218 parameters
4 restraints
H atoms treated by a mixture of independent and constrained refinement
Δρ_max_ = 0.41 e Å^−3^
Δρ_min_ = −0.48 e Å^−3^

### 

Data collection: *APEX2* (Bruker, 2008 ▶); cell refinement: *SAINT* (Bruker, 2008 ▶); data reduction: *SAINT*; program(s) used to solve structure: *SHELXS97* (Sheldrick, 2008 ▶); program(s) used to refine structure: *SHELXL97* (Sheldrick, 2008 ▶); molecular graphics: *ORTEP-3 for Windows* (Farrugia, 2012 ▶); software used to prepare material for publication: *SHELXL97* and *PLATON* (Spek, 2009 ▶).

## Supplementary Material

Crystal structure: contains datablock(s) global, I. DOI: 10.1107/S1600536813012774/su2597sup1.cif

Structure factors: contains datablock(s) I. DOI: 10.1107/S1600536813012774/su2597Isup2.hkl

Supplementary material file. DOI: 10.1107/S1600536813012774/su2597Isup3.cml

Additional supplementary materials:  crystallographic information; 3D view; checkCIF report

## Figures and Tables

**Table 1:** Hydrogen-bond geometry (Å, °) *Cg*1 is the centroid of the pyran ring (C7/C8/C13/O1/C14/C15).

*D*—H⋯*A*	*D*—H	H⋯*A*	*D*⋯*A*	*D*—H⋯*A*
N2—H2*A*⋯O3	0.90 (2)	1.89 (2)	2.595 (3)	134 (2)
C2—H2⋯O4^i^	0.93	2.55	3.442 (4)	162
C10—H10*B*⋯*Cg*1^ii^	0.97	2.77	3.527 (3)	136
C16—H16*B*⋯*Cg*1^iii^	0.96	2.73	3.606 (4)	153

Symmetry codes: (i) 
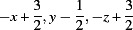
; (ii) 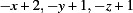; (iii) 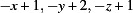.

**Fig. 1.**
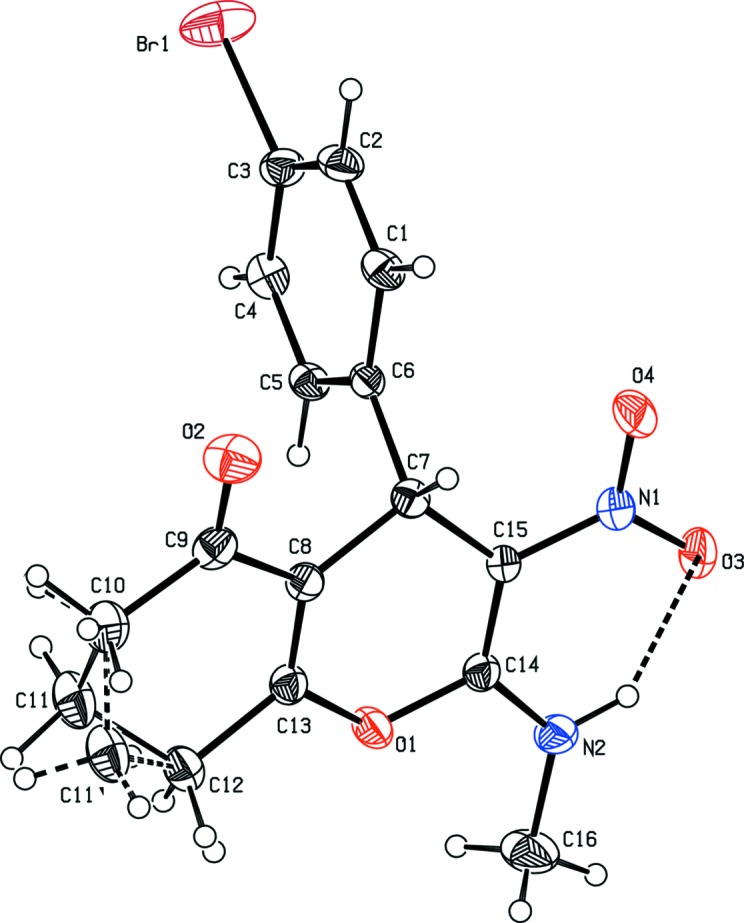
The molecular structure of the title molecule, with atom labelling. Displacement ellipsoids are drawn at 30% probability level. The intramolecular hydrogen bond, which generates an S(6) ring motif, is shown as a dashed line.

**Fig. 2.**
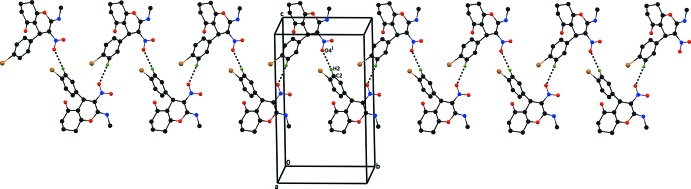
The crystal packing of the title compound, viewed along the c-axis, showing C2—H2···O4i hydrogen bonds resulting in the formation of C(8) chains running parallel to the b axis [hydrogen atoms not involved in the hydrogen bonding have been omitted for clarity; symmetry code: (i) -x+3/2, y+1/2, -z+1/2].

## supplementary crystallographic information

### Comment

Chromene derivatives are very important heterocyclic compounds that have a variety of industrial, biological and chemical synthesis applications (Geen *et al.*, 1996; Ercole *et al.*, 2009). They exhibit a number of pharmacological activities such as anti-HIV, anti-inflammatory, anti-bacterial, anti-allergic, anti-cancer, *etc*. (Khan *et al.*, 2010, Raj *et al.*, 2010). Against this background an X-ray diffraction study of the title compound and its structural aspects are presented herein.

The title compound, Fig. 1, consists of a chromene moiety attached to a chlorophenyl ring, a nitro group and a methylamine group. The molecular structure is stabilized by an intramolecular N—H···O hydrogen bonds, which generates an *S*(6) ring motif (Table 1 and Fig. 1). The methylene group carbon atom C11 of the chromene moiety is disordered over two positions (C11/C11') with an occupancy ratio of 0.791 (7): 0.209 (7). The pyran ring (C7/C8/C13-C15/O1) makes a dihedral angle of 87.11 (12) ° with the cholorophenyl ring (C1–C6), indicating that they are almost orthogonal.

The mean planes of the nitro and methylamine groups are almost co-planar with the pyran ring, with dihedral angles of 4.66 (20) and 3.87 (19) °, respectively. The mean plane of six membered carbocyclic ring (C8–C10/C11-C13) makes a dihedral angle of 86.50 (14) ° with the chlorophenyl ring, which shows that they too are almost perpendicular to each other.

The six membered carbocyclic ring (C8-C10/C11-C13) of the chromene moiety adopts an *envelope* conformation on C11 atom which deviates by 0.302 (4) Å out of the mean plane formed by the remaining ring atoms. The amine group nitrogen atom N2 deviates by -0.2382 (25) Å from the pyran ring. The bromine atom Br1 deviates from the phenyl ring (C1–C6) by 0.0953 (4) Å. The title compound exhibits structural similarities with a related structure (Sun *et al.*, 2012).

In the crystal, molecules are linked *via* C—H···O hydrogen bonds, which generate *C*(8) chains running parallel to the *b* axis (Bernstein *et al.*,1995); see Table 1 and Fig. 2. The crystal structure is further stabilized by C-H···π interactions (Table 1).

### Experimental

A solution of the 4-bromobenzaldehyde (0.18 g, 1.0 mmol), cyclic 1,3-dicarbonyl compound (1.0 mmol), NMSM (0.15 g, 1.0 mmol) and piperidine (0.2 equiv) in EtOH (2 ml) was stirred for 3.5 hrs. After the reaction was complete, as indicated by TLC, the product was filtered and washed with EtOH (2 ml) to remove excess base and other impurities. Single crystals suitable for X-ray diffraction were prepared by slow evaporation of a solution of the title compound in ethanol at room temperature.

### Refinement

The H atoms were localed from difference electron density maps and their distances were geometrically constrained. The amine group H atoms were constrained: N—H = 0.90 (1) Å with *U*_iso_(H) = 1.2*U*_eq_(N). The C-bound H atoms were treated as riding atoms: C—H = 0.93, 0.97, 0.96 and 0.98 Å for CH(aromatic), methylene, methine and methyl H atoms, respectively, with *U*_iso_(H) = k × *U*_eq_(C) where k = 1.5 for methyl H atoms and = 1.2 for other H atoms. The rotation angles for the methyl groups were optimized by least squares. The bond distances of the disordered components of atom C11 were restrained using standard similarity restraint SADI [*SHELXL97*, Sheldrick, 2008] with s.u. of 0.01 Å. The atomic displacement parameters of the major and minor components were made equal using the constraint EADP.

### Crystal data

**Table:**

C_16_H_15_BrN_2_O_4_	*F*(000) = 768
*M**_r_* = 379.18	*D*_x_ = 1.575 Mg m^−^^3^
Monoclinic, *P*2_1_/*n*	Mo *K*α radiation, λ = 0.71073 Å
Hall symbol: -P 2yn	Cell parameters from 2053 reflections
*a* = 8.1114 (9) Å	θ = 2.2–26.0°
*b* = 10.8530 (13) Å	µ = 2.59 mm^−^^1^
*c* = 18.222 (2) Å	*T* = 296 K
β = 94.399 (6)°	Block, colourless
*V* = 1599.4 (3) Å^3^	0.30 × 0.25 × 0.25 mm
*Z* = 4	

### Data collection

**Table:**

Bruker SMART APEXII CCD diffractometer	3130 independent reflections
Radiation source: fine-focus sealed tube	2053 reflections with *I* > 2σ(*I*)
Graphite monochromator	*R*_int_ = 0.047
ω and φ scans	θ_max_ = 26.0°, θ_min_ = 2.2°
Absorption correction: multi-scan (*SADABS*; Bruker, 2008)	*h* = −10→10
*T*_min_ = 0.464, *T*_max_ = 0.523	*k* = −12→13
12198 measured reflections	*l* = −19→22

### Refinement

**Table:**

Refinement on *F*^2^	Primary atom site location: structure-invariant direct methods
Least-squares matrix: full	Secondary atom site location: difference Fourier map
*R*[*F*^2^ > 2σ(*F*^2^)] = 0.039	Hydrogen site location: inferred from neighbouring sites
*wR*(*F*^2^) = 0.101	H atoms treated by a mixture of independent and constrained refinement
*S* = 1.04	*w* = 1/[σ^2^(*F*_o_^2^) + (0.047*P*)^2^ + 0.3202*P*] where *P* = (*F*_o_^2^ + 2*F*_c_^2^)/3
3130 reflections	(Δ/σ)_max_ = 0.002
218 parameters	Δρ_max_ = 0.41 e Å^−^^3^
4 restraints	Δρ_min_ = −0.48 e Å^−^^3^

### Special details

**Table:**

Geometry. All e.s.d.'s (except the e.s.d. in the dihedral angle between two l.s. planes) are estimated using the full covariance matrix. The cell e.s.d.'s are taken into account individually in the estimation of e.s.d.'s in distances, angles and torsion angles; correlations between e.s.d.'s in cell parameters are only used when they are defined by crystal symmetry. An approximate (isotropic) treatment of cell e.s.d.'s is used for estimating e.s.d.'s involving l.s. planes.
Refinement. Refinement of *F*^2^ against ALL reflections. The weighted *R*-factor *wR* and goodness of fit *S* are based on *F*^2^, conventional *R*-factors *R* are based on *F*, with *F* set to zero for negative *F*^2^. The threshold expression of *F*^2^ > σ(*F*^2^) is used only for calculating *R*-factors(gt) *etc*. and is not relevant to the choice of reflections for refinement. *R*-factors based on *F*^2^ are statistically about twice as large as those based on *F*, and *R*- factors based on ALL data will be even larger.

### Fractional atomic coordinates and isotropic or equivalent isotropic displacement parameters (Å^2^)

**Table:**

	*x*	*y*	*z*	*U*_iso_*/*U*_eq_	Occ. (<1)
C5	0.6240 (3)	0.6938 (2)	0.54373 (14)	0.0310 (6)	
H5	0.5953	0.7167	0.4952	0.037*	
C4	0.5278 (3)	0.6093 (3)	0.57717 (15)	0.0359 (7)	
H4	0.4339	0.5763	0.5520	0.043*	
C3	0.5733 (4)	0.5744 (3)	0.64890 (16)	0.0394 (7)	
C2	0.7093 (3)	0.6247 (3)	0.68770 (15)	0.0386 (7)	
H2	0.7379	0.6011	0.7361	0.046*	
C1	0.8019 (3)	0.7104 (3)	0.65351 (14)	0.0359 (7)	
H1	0.8930	0.7458	0.6796	0.043*	
C6	0.7628 (3)	0.7455 (2)	0.58089 (13)	0.0279 (6)	
C7	0.8703 (3)	0.8371 (2)	0.54267 (14)	0.0297 (6)	
H7	0.9625	0.8614	0.5776	0.036*	
C8	0.9402 (3)	0.7776 (2)	0.47729 (14)	0.0305 (6)	
C9	1.0663 (3)	0.6799 (3)	0.49073 (17)	0.0384 (7)	
C10	1.1286 (4)	0.6155 (3)	0.42605 (18)	0.0543 (9)	
H10A	1.2327	0.6526	0.4151	0.065*	0.791 (7)
H10B	1.1504	0.5300	0.4391	0.065*	0.791 (7)
H10C	1.0740	0.5360	0.4218	0.065*	0.208 (7)
H10D	1.2456	0.5997	0.4374	0.065*	0.208 (7)
C11	1.0137 (6)	0.6194 (4)	0.3590 (2)	0.0529 (13)	0.791 (7)
H11A	0.9204	0.5658	0.3657	0.063*	0.791 (7)
H11B	1.0699	0.5882	0.3177	0.063*	0.791 (7)
C11'	1.1058 (18)	0.6734 (16)	0.3518 (6)	0.0529 (13)	0.208 (7)
H11C	1.1024	0.6091	0.3147	0.063*	0.208 (7)
H11D	1.2005	0.7254	0.3445	0.063*	0.208 (7)
C12	0.9494 (4)	0.7503 (3)	0.34074 (15)	0.0441 (8)	
H12A	1.0366	0.7993	0.3216	0.053*	0.791 (7)
H12B	0.8578	0.7463	0.3034	0.053*	0.791 (7)
H12C	0.9658	0.8145	0.3050	0.053*	0.208 (7)
H12D	0.8621	0.6967	0.3201	0.053*	0.208 (7)
C13	0.8943 (3)	0.8086 (2)	0.40842 (15)	0.0331 (6)	
C14	0.7371 (3)	0.9798 (2)	0.44461 (15)	0.0314 (6)	
C15	0.7778 (3)	0.9516 (2)	0.51749 (14)	0.0296 (6)	
C16	0.6219 (5)	1.1042 (4)	0.33983 (17)	0.0643 (10)	
H16A	0.7247	1.1215	0.3191	0.096*	
H16B	0.5510	1.1749	0.3342	0.096*	
H16C	0.5694	1.0351	0.3149	0.096*	
N1	0.7303 (3)	1.0296 (2)	0.57208 (14)	0.0391 (6)	
N2	0.6532 (3)	1.0757 (2)	0.41753 (13)	0.0413 (6)	
O1	0.7851 (2)	0.90458 (17)	0.39070 (10)	0.0401 (5)	
O2	1.1199 (2)	0.6558 (2)	0.55357 (12)	0.0550 (6)	
O3	0.6431 (3)	1.12423 (18)	0.55655 (11)	0.0497 (6)	
O4	0.7744 (3)	1.0045 (2)	0.63710 (12)	0.0555 (6)	
Br1	0.44896 (5)	0.45158 (4)	0.69353 (2)	0.0806 (2)	
H2A	0.616 (4)	1.124 (2)	0.4528 (13)	0.058 (10)*	

### Atomic displacement parameters (Å^2^)

**Table:**

	*U*^11^	*U*^22^	*U*^33^	*U*^12^	*U*^13^	*U*^23^
C5	0.0327 (15)	0.0365 (16)	0.0231 (14)	0.0076 (13)	−0.0023 (11)	0.0015 (12)
C4	0.0264 (15)	0.0419 (17)	0.0391 (18)	0.0027 (13)	−0.0004 (12)	−0.0038 (14)
C3	0.0394 (17)	0.0393 (17)	0.0411 (18)	0.0045 (14)	0.0135 (14)	0.0076 (14)
C2	0.0438 (18)	0.0467 (18)	0.0254 (15)	0.0066 (15)	0.0031 (13)	0.0056 (13)
C1	0.0355 (16)	0.0449 (17)	0.0265 (16)	0.0022 (14)	−0.0034 (12)	−0.0029 (13)
C6	0.0324 (15)	0.0278 (14)	0.0235 (14)	0.0050 (12)	0.0017 (11)	−0.0006 (11)
C7	0.0284 (14)	0.0322 (15)	0.0276 (15)	−0.0010 (12)	−0.0036 (11)	−0.0012 (12)
C8	0.0284 (14)	0.0301 (15)	0.0335 (16)	0.0001 (12)	0.0044 (12)	0.0002 (12)
C9	0.0311 (15)	0.0361 (17)	0.049 (2)	0.0002 (13)	0.0076 (14)	0.0054 (15)
C10	0.058 (2)	0.046 (2)	0.060 (2)	0.0154 (17)	0.0153 (18)	−0.0003 (17)
C11	0.051 (3)	0.053 (3)	0.054 (2)	0.014 (2)	0.004 (2)	−0.015 (2)
C11'	0.051 (3)	0.053 (3)	0.054 (2)	0.014 (2)	0.004 (2)	−0.015 (2)
C12	0.0520 (19)	0.0428 (18)	0.0384 (18)	0.0071 (15)	0.0094 (14)	−0.0051 (14)
C13	0.0334 (15)	0.0300 (15)	0.0363 (17)	0.0019 (13)	0.0063 (12)	−0.0008 (13)
C14	0.0324 (15)	0.0282 (15)	0.0344 (17)	0.0013 (13)	0.0069 (12)	0.0002 (13)
C15	0.0364 (15)	0.0252 (14)	0.0275 (16)	−0.0003 (12)	0.0042 (12)	−0.0021 (12)
C16	0.082 (3)	0.073 (2)	0.038 (2)	0.035 (2)	0.0092 (17)	0.0189 (18)
N1	0.0488 (15)	0.0331 (15)	0.0356 (16)	−0.0028 (12)	0.0041 (12)	−0.0048 (12)
N2	0.0505 (15)	0.0375 (15)	0.0365 (15)	0.0126 (12)	0.0082 (12)	0.0085 (12)
O1	0.0508 (12)	0.0419 (11)	0.0278 (10)	0.0177 (10)	0.0041 (9)	0.0000 (9)
O2	0.0472 (13)	0.0663 (15)	0.0507 (15)	0.0186 (11)	−0.0005 (11)	0.0140 (12)
O3	0.0660 (15)	0.0338 (12)	0.0497 (13)	0.0142 (11)	0.0072 (11)	−0.0046 (10)
O4	0.0869 (17)	0.0503 (13)	0.0282 (13)	0.0066 (12)	−0.0027 (11)	−0.0077 (10)
Br1	0.0731 (3)	0.0910 (4)	0.0789 (3)	−0.0277 (2)	0.0142 (2)	0.0337 (2)

### Geometric parameters (Å, º)

**Table:**

C5—C4	1.376 (4)	C10—H10D	0.9700
C5—C6	1.387 (3)	C11—C12	1.541 (5)
C5—H5	0.9300	C11—H11A	0.9700
C4—C3	1.384 (4)	C11—H11B	0.9700
C4—H4	0.9300	C11'—C12	1.519 (9)
C3—C2	1.376 (4)	C11'—H11C	0.9700
C3—Br1	1.893 (3)	C11'—H11D	0.9700
C2—C1	1.375 (4)	C12—C13	1.485 (4)
C2—H2	0.9300	C12—H12A	0.9700
C1—C6	1.390 (3)	C12—H12B	0.9700
C1—H1	0.9300	C12—H12C	0.9700
C6—C7	1.526 (4)	C12—H12D	0.9700
C7—C8	1.504 (4)	C13—O1	1.389 (3)
C7—C15	1.504 (4)	C14—N2	1.318 (3)
C7—H7	0.9800	C14—O1	1.357 (3)
C8—C13	1.325 (4)	C14—C15	1.378 (4)
C8—C9	1.480 (4)	C15—N1	1.383 (3)
C9—O2	1.222 (3)	C16—N2	1.452 (4)
C9—C10	1.492 (4)	C16—H16A	0.9600
C10—C11	1.480 (5)	C16—H16B	0.9600
C10—C11'	1.491 (9)	C16—H16C	0.9600
C10—H10A	0.9700	N1—O4	1.241 (3)
C10—H10B	0.9700	N1—O3	1.267 (3)
C10—H10C	0.9700	N2—H2A	0.897 (10)
			
C4—C5—C6	121.3 (2)	H10C—C11—H11A	75.8
C4—C5—H5	119.3	C10—C11—H11B	109.0
C6—C5—H5	119.3	C12—C11—H11B	109.0
C5—C4—C3	118.8 (3)	H10C—C11—H11B	103.5
C5—C4—H4	120.6	H11A—C11—H11B	107.8
C3—C4—H4	120.6	C10—C11'—C12	113.3 (7)
C2—C3—C4	121.5 (3)	C10—C11'—H11C	108.9
C2—C3—Br1	119.4 (2)	C12—C11'—H11C	108.9
C4—C3—Br1	119.1 (2)	C10—C11'—H11D	108.9
C3—C2—C1	118.7 (3)	C12—C11'—H11D	108.9
C3—C2—H2	120.7	H11C—C11'—H11D	107.7
C1—C2—H2	120.7	C13—C12—C11'	115.2 (5)
C2—C1—C6	121.6 (3)	C13—C12—C11	109.4 (3)
C2—C1—H1	119.2	C13—C12—H12A	109.8
C6—C1—H1	119.2	C11'—C12—H12A	74.0
C5—C6—C1	118.2 (2)	C11—C12—H12A	109.8
C5—C6—C7	120.8 (2)	C13—C12—H12B	109.8
C1—C6—C7	121.1 (2)	C11'—C12—H12B	131.0
C8—C7—C15	108.8 (2)	C11—C12—H12B	109.8
C8—C7—C6	110.2 (2)	H12A—C12—H12B	108.2
C15—C7—C6	112.9 (2)	C13—C12—H12C	108.5
C8—C7—H7	108.3	C11'—C12—H12C	109.1
C15—C7—H7	108.3	C11—C12—H12C	138.2
C6—C7—H7	108.3	H12B—C12—H12C	72.4
C13—C8—C9	118.7 (2)	C13—C12—H12D	108.7
C13—C8—C7	123.0 (2)	C11'—C12—H12D	107.4
C9—C8—C7	118.3 (2)	C11—C12—H12D	75.9
O2—C9—C8	120.0 (3)	H12A—C12—H12D	136.0
O2—C9—C10	121.5 (3)	H12C—C12—H12D	107.6
C8—C9—C10	118.5 (3)	C8—C13—O1	122.6 (2)
C11—C10—C9	114.1 (3)	C8—C13—C12	126.7 (3)
C11'—C10—C9	119.6 (6)	O1—C13—C12	110.7 (2)
C11—C10—H10A	108.7	N2—C14—O1	111.9 (2)
C11'—C10—H10A	71.8	N2—C14—C15	128.0 (2)
C9—C10—H10A	108.7	O1—C14—C15	120.2 (2)
C11—C10—H10B	108.7	C14—C15—N1	119.8 (2)
C11'—C10—H10B	129.3	C14—C15—C7	123.7 (2)
C9—C10—H10B	108.7	N1—C15—C7	116.5 (2)
H10A—C10—H10B	107.6	N2—C16—H16A	109.5
C11—C10—H10C	72.8	N2—C16—H16B	109.5
C11'—C10—H10C	106.1	H16A—C16—H16B	109.5
C9—C10—H10C	107.4	N2—C16—H16C	109.5
H10A—C10—H10C	138.8	H16A—C16—H16C	109.5
C11—C10—H10D	136.4	H16B—C16—H16C	109.5
C11'—C10—H10D	108.6	O4—N1—O3	120.4 (2)
C9—C10—H10D	107.5	O4—N1—C15	118.4 (2)
H10B—C10—H10D	67.9	O3—N1—C15	121.2 (2)
H10C—C10—H10D	107.1	C14—N2—C16	125.4 (3)
C10—C11—C12	112.7 (3)	C14—N2—H2A	112 (2)
C12—C11—H10C	143.2	C16—N2—H2A	122 (2)
C10—C11—H11A	109.0	C14—O1—C13	119.7 (2)
C12—C11—H11A	109.0		
			
C6—C5—C4—C3	−1.1 (4)	C10—C11'—C12—C13	30.1 (16)
C5—C4—C3—C2	1.8 (4)	C10—C11'—C12—C11	−58.9 (8)
C5—C4—C3—Br1	−176.51 (19)	C10—C11—C12—C13	−47.2 (4)
C4—C3—C2—C1	−0.7 (4)	C10—C11—C12—C11'	59.3 (8)
Br1—C3—C2—C1	177.6 (2)	C9—C8—C13—O1	−175.7 (2)
C3—C2—C1—C6	−1.0 (4)	C7—C8—C13—O1	4.3 (4)
C4—C5—C6—C1	−0.6 (4)	C9—C8—C13—C12	3.7 (4)
C4—C5—C6—C7	178.8 (2)	C7—C8—C13—C12	−176.3 (3)
C2—C1—C6—C5	1.7 (4)	C11'—C12—C13—C8	−17.8 (9)
C2—C1—C6—C7	−177.7 (2)	C11—C12—C13—C8	21.6 (4)
C5—C6—C7—C8	−60.6 (3)	C11'—C12—C13—O1	161.7 (8)
C1—C6—C7—C8	118.7 (3)	C11—C12—C13—O1	−158.9 (3)
C5—C6—C7—C15	61.2 (3)	N2—C14—C15—N1	−0.6 (4)
C1—C6—C7—C15	−119.4 (3)	O1—C14—C15—N1	179.2 (2)
C15—C7—C8—C13	−13.3 (3)	N2—C14—C15—C7	178.1 (3)
C6—C7—C8—C13	110.9 (3)	O1—C14—C15—C7	−2.1 (4)
C15—C7—C8—C9	166.7 (2)	C8—C7—C15—C14	12.3 (3)
C6—C7—C8—C9	−69.1 (3)	C6—C7—C15—C14	−110.3 (3)
C13—C8—C9—O2	174.6 (3)	C8—C7—C15—N1	−168.9 (2)
C7—C8—C9—O2	−5.4 (4)	C6—C7—C15—N1	68.4 (3)
C13—C8—C9—C10	−3.6 (4)	C14—C15—N1—O4	−177.1 (2)
C7—C8—C9—C10	176.4 (2)	C7—C15—N1—O4	4.1 (4)
O2—C9—C10—C11	158.4 (3)	C14—C15—N1—O3	3.3 (4)
C8—C9—C10—C11	−23.5 (4)	C7—C15—N1—O3	−175.6 (2)
O2—C9—C10—C11'	−159.3 (9)	O1—C14—N2—C16	−3.4 (4)
C8—C9—C10—C11'	18.8 (9)	C15—C14—N2—C16	176.4 (3)
C11'—C10—C11—C12	−58.5 (7)	N2—C14—O1—C13	170.9 (2)
C9—C10—C11—C12	49.3 (5)	C15—C14—O1—C13	−8.9 (4)
C11—C10—C11'—C12	60.3 (9)	C8—C13—O1—C14	8.0 (4)
C9—C10—C11'—C12	−31.7 (17)	C12—C13—O1—C14	−171.5 (2)

### Hydrogen-bond geometry (Å, º)

Cg1 is the centroid of the pyran ring (C7/C8/C13/O1/C14/C15).

**Table:**

*D*—H···*A*	*D*—H	H···*A*	*D*···*A*	*D*—H···*A*
N2—H2*A*···O3	0.90 (2)	1.89 (2)	2.595 (3)	134 (2)
C2—H2···O4^i^	0.93	2.55	3.442 (4)	162
C10—H10*B*···*Cg*1^ii^	0.97	2.77	3.527 (3)	136
C16—H16*B*···*Cg*1^iii^	0.96	2.73	3.606 (4)	153

Symmetry codes: (i) −*x*+3/2, *y*−1/2, −*z*+3/2; (ii) −*x*+2, −*y*+1, −*z*+1; (iii) −*x*+1, −*y*+2, −*z*+1.
